# Identification and Characterization of Cancer Mutations in Japanese Lung Adenocarcinoma without Sequencing of Normal Tissue Counterparts

**DOI:** 10.1371/journal.pone.0073484

**Published:** 2013-09-12

**Authors:** Ayako Suzuki, Sachiyo Mimaki, Yuki Yamane, Akikazu Kawase, Koutatsu Matsushima, Makito Suzuki, Koichi Goto, Sumio Sugano, Hiroyasu Esumi, Yutaka Suzuki, Katsuya Tsuchihara

**Affiliations:** 1 Department of Medical Genome Sciences, Graduate School of Frontier Sciences, The University of Tokyo, Chiba, Japan; 2 Division of Translational Research, Research Center for Innovative Oncology, National Cancer Center Hospital East, Chiba, Japan; 3 Thoracic Oncology Division, National Cancer Center Hospital East, Chiba, Japan; Institute of Molecular Medicine, Taiwan

## Abstract

We analyzed whole-exome sequencing data from 97 Japanese lung adenocarcinoma patients and identified several putative cancer-related genes and pathways. Particularly, we observed that cancer-related mutation patterns were significantly different between different ethnic groups. As previously reported, mutations in the EGFR gene were characteristic to Japanese, while those in the KRAS gene were more frequent in Caucasians. Furthermore, during the course of this analysis, we found that cancer-specific somatic mutations can be detected without sequencing normal tissue counterparts. 64% of the germline variants could be excluded using a total of 217 external Japanese exome datasets. We also show that a similar approach may be used for other three ethnic groups, although the discriminative power depends on the ethnic group. We demonstrate that the ATM gene and the PAPPA2 gene could be identified as cancer prognosis related genes. By bypassing the sequencing of normal tissue counterparts, this approach provides a useful means of not only reducing the time and cost of sequencing but also analyzing archive samples, for which normal tissue counterparts are not available.

## Introduction

The advent of next generation sequencing technology has greatly facilitated the detection and characterization of genetic variations in the human genome. Most remarkably, this type of study has driven the 1000 Genomes Project [1,2], which aims to provide a comprehensive map of human genetic variants across various ethnic backgrounds. However, because whole-genome sequencing is still costly, the sequencing of whole exon regions using hybridization capture methods (exome sequencing) [3-5] is widely used to screen for genes that are related to hereditary diseases. By sequencing exomes from healthy and diseased individuals and comparing them, genes that are responsible for many diseases have been identified [6], including Miller syndrome [7,8] and familial hyperkalemic hypertension [9]. Along with the progress that has been made in exome sequencing, the volume of germline single nucleotide polymorphism (SNP) data that has been registered in dbSNP is rapidly expanding for various populations [10].

Exome sequencing provides a powerful tool for cancer studies as well. Indeed, a number of papers have been published describing the identification and characterization of single nucleotide variants (SNVs) that somatically occur in cancers and are suspected to be responsible for carcinogenesis and disease development [11]. The International Cancer Genome Consortium (ICGC) has been collecting exome data for somatic SNVs that are present in more than 50 types of cancers as a part of an international collaborative effort [12-14]. The Cancer Genome Atlas (TCGA) has developed a large genomic dataset, including exomes for high-grade ovarian carcinoma, that has been used to detect significantly mutated genes, including TP53, BRCA1 and BRCA2 [15]. They have also identified various genomic aberrations and deregulated pathways that may act as therapeutic targets.

In most ongoing cancer exome studies, normal tissue counterparts have been sequenced in parallel with cancer tissue [15-19]. This is assumed to be necessary because germline variants must be excluded from the full set of SNVs to detect the somatic SNVs that are unique to cancers. However, the sequencing of normal tissue counterparts increases the cost and time of the analysis. Also, in some cases, it is difficult to obtain normal tissue counterparts. In addition, it remains unclear how accurately germline SNVs can be excluded using normal tissue exomes. To conservatively exclude germline SNVs, their sequence depths and accuracies may need to be greater than those that are obtained from the cancer exomes.

In this study, we generated and analyzed 97 cancer exomes from Japanese lung adenocarcinoma patients. We also demonstrate that somatic SNVs can be enriched to a level that is sufficient for further statistical analyses even in the absence of the sequencing of normal tissue counterparts. To separate the germline from the somatic SNVs, we first compared the variation patterns between a cancer exome with the 96 other patients’ normal tissue exomes. We also attempted to conduct a similar mutual comparison solely utilizing cancer exomes, without the consideration of exomes of normal tissue counterparts. It is true that if we completely omitted normal tissue sequencing, we would tentatively disregard of somatic mutations that occurs at exactly the same genomic position in multiple cancers. However, recent papers have elucidated that such shared SNVs are very rare [15,20-22]. Moreover, many of these recursively mutations have been registered in the cancer somatic mutation databases such as Sanger COSMIC [23,24], and those recurrent SNVs can be recovered by follow-up studies partially using the data from the normal tissues. To understand the unique nature of each cancer, a statistical analysis of the distinct SNVs is presumed to be essential in addition to the analysis of the common SNVs.

In this study, we demonstrate that it is possible to identify the first candidates for cancer-related genes and pathways, even without the sequencing of a normal tissue counterpart. We show that this approach is useful not only to reduce the cost of the sequencing but also to improve the fidelity of the data. It should be also useful for analyzing old archive samples, for which normal tissue counterparts are not always available. Here, we describe a practical and cost-effective method to expedite cancer exome sequencing. 

## Results and Discussion

### Characterization of SNVs using the 97 exome dataset

Firstly, we generated and analyzed whole-exome sequences from 97 Japanese lung adenocarcinoma patients. Exome data were collected from both cancer and normal-tissue counterparts, separated by laser capture microdissection. We purified the exonic DNA (exomes) and generated 76-base paired-end reads using the illumina GAIIx platform. Approximately 30 million mapped sequences were obtained from each sample, providing 74× coverage of the target regions; 93% of the target regions had 5× coverage (Figure S1 in File **S1**). Burrows-Wheeler Aligner (BWA) [25] and the Genome Analysis Toolkit (GATK) [26,27] were used to identify SNVs (Figure S2 in File **S1**). Only SNVs that were detected in cancer tissues and showed no evidence of variation in normal tissues were selected for further analysis.

**Figure 1:**
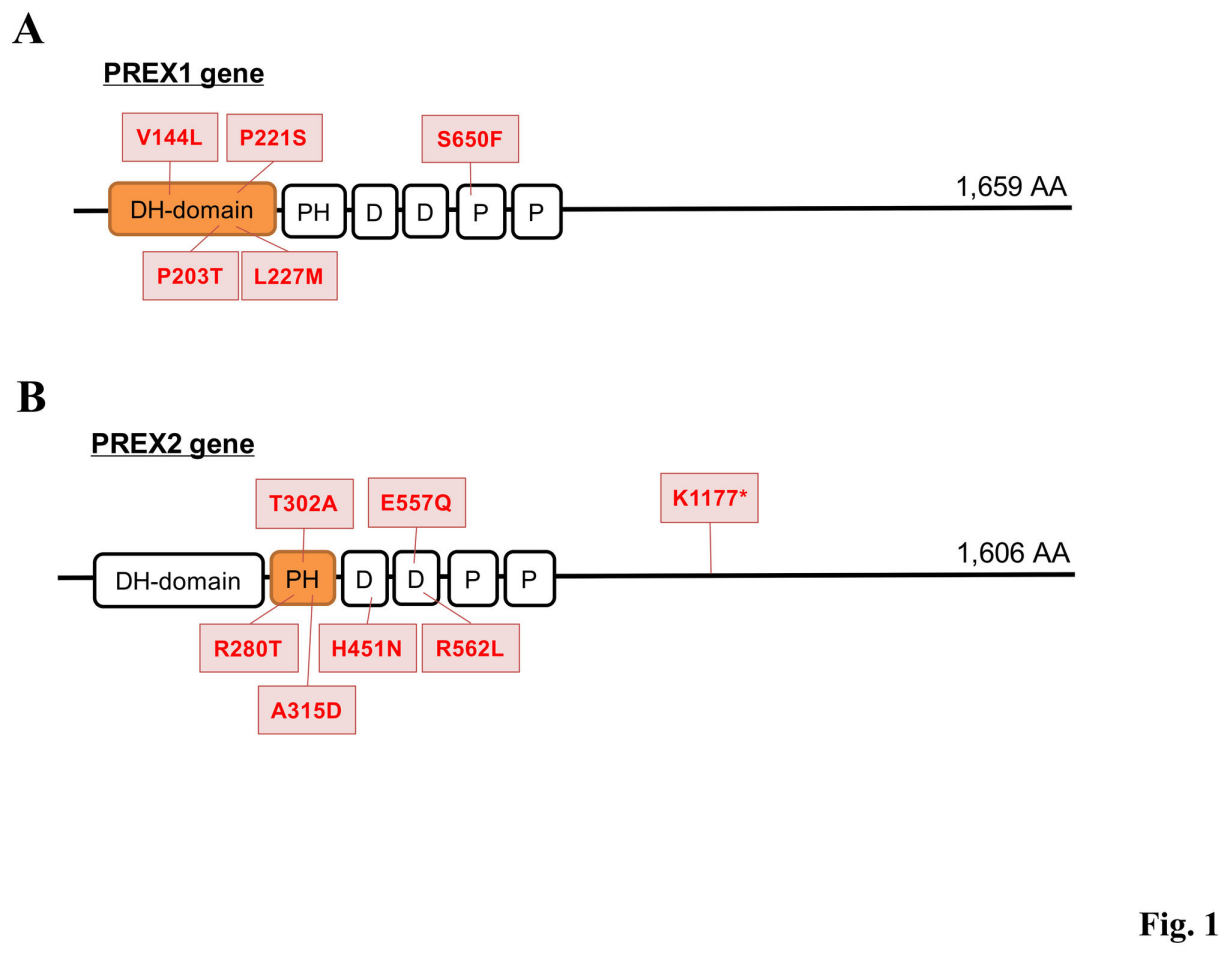
Identification and characterization of the putative cancer-related genes using 97 cancer exomes. SNVs in the PREX1 (**A**) and PREX2 (**B**) genes are represented in the boxes. The protein domains in which the enrichments of the SNVs were statistically significant are represented in orange boxes (also see Materials and Method). DH-domain: Dbl homology (DH) domain; PH: Pleckstrin homology domain; D: DEP domain; P: PDZ/DHR/GLGF.

The obtained dataset was used to characterize the cancer-specific mutation patterns (Table S3 in File **S1**). We calculated the enrichment of the SNVs within particular genes, protein domains, functional categories, and pathways. We searched for genes with somatic SNVs significantly enriched in Japanese lung adenocarcinoma. As shown in Table S4 in File **S1**, several genes were identified as significantly mutated. In particular, we searched for domains that are enriched with SNVs and harbor known cancer-related mutations in the COSMIC database. In total, 11 genes were identified (P < 0.02, Table **1**). For example, the Dbl homology (DH) domain of PREX1 gene [28] was enriched with SNVs (*P* = 0.00071). However, in the PREX2 gene [29], the Pleckstrin homology (PH) domain was enriched with SNVs (*P* = 0.011) (Figure 1A and B). Both the PREX1 and the PREX2 genes activate the exchange of GDP to GTP for the Rho family of GTPases and the DH/PH domains are indispensable for nucleotide exchange of GTPases and its regulation [30-32]. In addition, we analyzed the expression patterns of these genes using a cancer gene expression database, GeneLogic (Figure S3 in File **S1**). Expression levels of PREX1 and PREX2 were not enhanced in lung adenocarcinoma but were enhanced in wide variety of cancers, which is partly indicated in previous studies [33]. The SNVs in the PREX1 and PREX2 genes, which were concentrated at its pivotal signaling domains, might enhance activities in these genes, and thereby functionally mimics the increased expressions of this gene in some different types of cancers. The cancer-related gene candidates identified from this dataset are listed in Table **1**.

**Table 1:** List of the identified possible cancer-related genes.

		Number of SNVs		
Gene	Domain	Domain	Gene	P-value^*^
EGFR^†^	IPR001245:Serine-threonine/tyrosine-protein kinase	34	37	4.4e-21
KRAS^†^	IPR001806:Ras GTPase	6	7	8.0e-6
TNN	IPR003961:Fibronectin, type III	4	5	5.2e-5
TP53^†^	IPR008967:p53-like transcription factor, DNA-binding	20	23	9.5e-5
PREX1	IPR000219:Dbl homology (DH) domain	4	5	0.00071
DNAH7	IPR004273:Dynein heavy chain	5	7	0.0025
FSTL5	IPR011044:Quinoprotein amine dehydrogenase, beta chain-like	7	7	0.0043
NRXN3	IPR008985:Concanavalin A-like lectin/glucanase	5	7	0.0063
PREX2	IPR001849:Pleckstrin homology	3	7	0.011
FER1L6	IPR008973:C2 calcium/lipid-binding domain, CaLB	3	6	0.013
COL22A	IPR008985:Concanavalin A-like lectin/glucanase	3	6	0.015

^*^ *P* < 0.02

^†^ Reported in the Cancer Gene Census [11]. Note that the genes atop the list are previously reported to be associated with this cancer type, while most of them are novel possible cancer-related genes.

**Figure 2:**
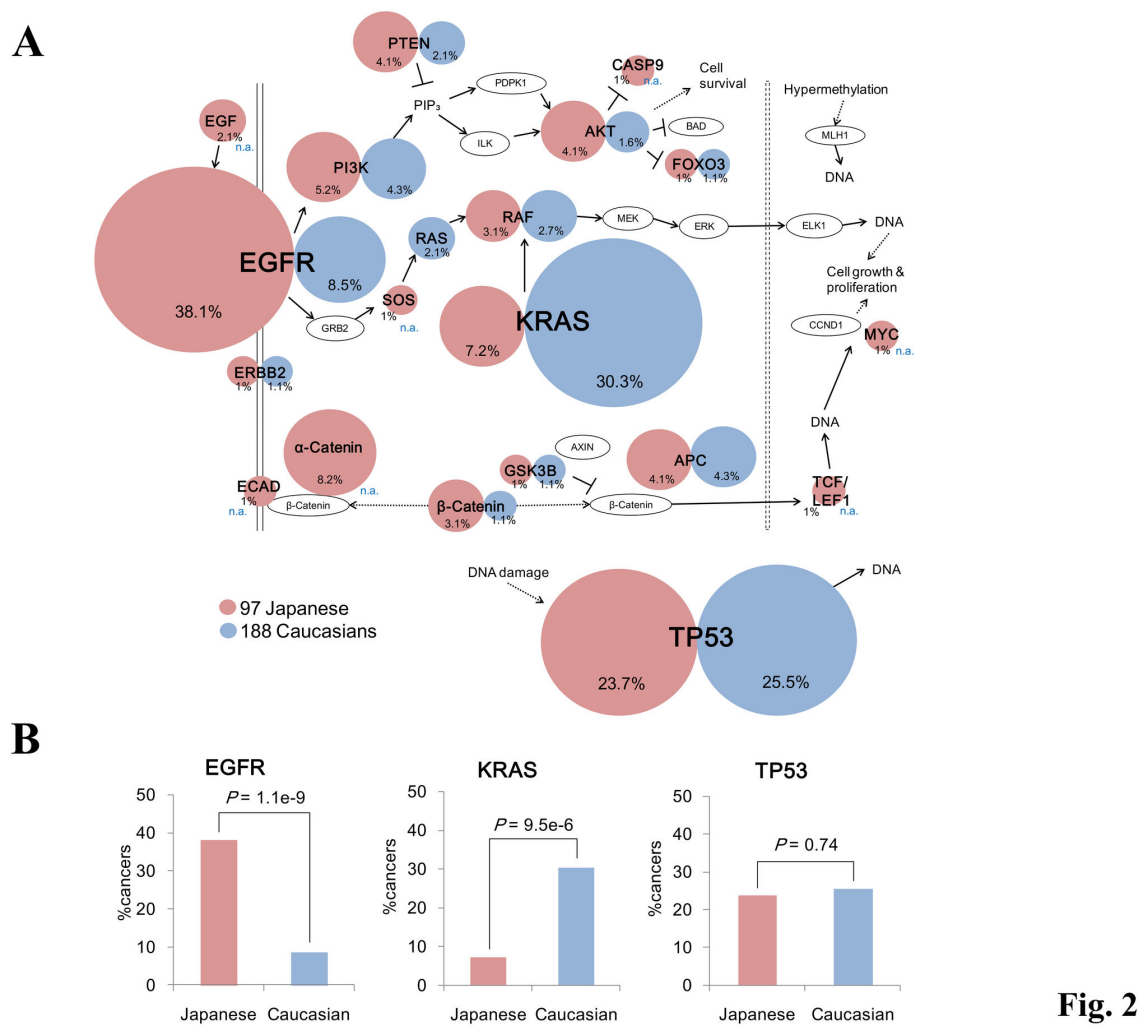
The EGFR/Ras pathways in Japanese and Caucasian populations. (**A**) Mutation patterns in the endometrial cancer pathway that was detected in the enrichment analysis are shown. The size of the circle represents the population of the cancers harboring the SNVs in the corresponding gene (percentage is also shown in the margin). SNVs in this study and the external dataset in Caucasian populations are shown in red and blue circles, respectively. n.a.: mutation frequencies were not available. (**B**) Comparison of mutation ratio of EGFR, KRAS and TP53 genes among both datasets. The p-values were calculated by two-sample test for equality of proportions.

Similarly, pathway enrichment analyses using the KEGG database [34] also detected several putative cancer-related pathways. The identified pathways are listed in Table **2**. Interestingly, the endometrial cancer pathway [35] was detected in this enrichment analysis (*P* = 3.1e-15, Figure **2A**). This pathway includes major cancer-related pathways, for example, the MAPK signaling pathway and the PI3K/AKT pathway. For this pathway, we compared mutation patterns between our Japanese data and those of the previous study of lung adenocarcinoma in Caucasians [21]. We found that the SNVs in the EGFR gene were four times more frequent in the Japanese population than among Caucasian populations (Figure **2B**, left panel). EGFR mutations were frequently occurring in non-smoker, female and Asian patients of lung adenocarcinoma [36], which is a molecular target of anti-cancer drug, *gefitinib* [20,37,38]. Conversely, KRAS mutations, which are also well-known cancer-related mutations [39], were more than four times frequent among Caucasians (Figure **2B**, center panel). However not all mutational patterns are different between populations. For instance, TP53 harbored mutations in both datasets with similar frequency (Figure **2B**, right panel).

**Table 2:** List of the identified possible cancer-related pathways.

KEGG ID	Pathway definition	Number of cancers with SNVs	P-value^*^
hsa05213	Endometrial cancer	72	3.1e-15
hsa04320	Dorso-ventral axis formation	48	4.4e-15
hsa05219	Bladder cancer	62	4.9e-14
hsa05223	Non-small cell lung cancer	66	7.1e-12
hsa05214	Glioma	70	6.5e-11
hsa05218	Melanoma	70	1.3e-9
hsa05212	Pancreatic cancer	68	6.9e-9
hsa05215	Prostate cancer	71	4.3e-7
hsa05216	Thyroid cancer	36	1.1e-6
hsa04520	Adherens junction	59	3.7e-6
hsa05210	Colorectal cancer	53	1.8e-5
hsa04012	ErbB signaling pathway	64	2.6e-5
hsa05120	Epithelial cell signaling in *Helicobacter pylori* infection	53	4.8e-5
hsa04540	Gap junction	60	0.00024
hsa04912	GnRH signaling pathway	61	0.0011
hsa05217	Basal cell carcinoma	41	0.0020
hsa05222	Small cell lung cancer	52	0.0069
hsa05220	Chronic myeloid leukemia	46	0.010
hsa05160	Hepatitis C	67	0.012
hsa05014	Amyotrophic lateral sclerosis (ALS)	36	0.014
hsa04977	Vitamin digestion and absorption	20	0.015
hsa05416	Viral myocarditis	40	0.028
hsa04512	ECM-receptor interaction	47	0.034
hsa02010	ABC transporters	29	0.035
hsa04510	Focal adhesion	78	0.037
hsa05412	Arrhythmogenic right ventricular cardiomyopathy (ARVC)	40	0.039

^*^ *P* < 0.05

### Ambiguity in SNV identification of normal tissue counterparts

**Figure 3:**
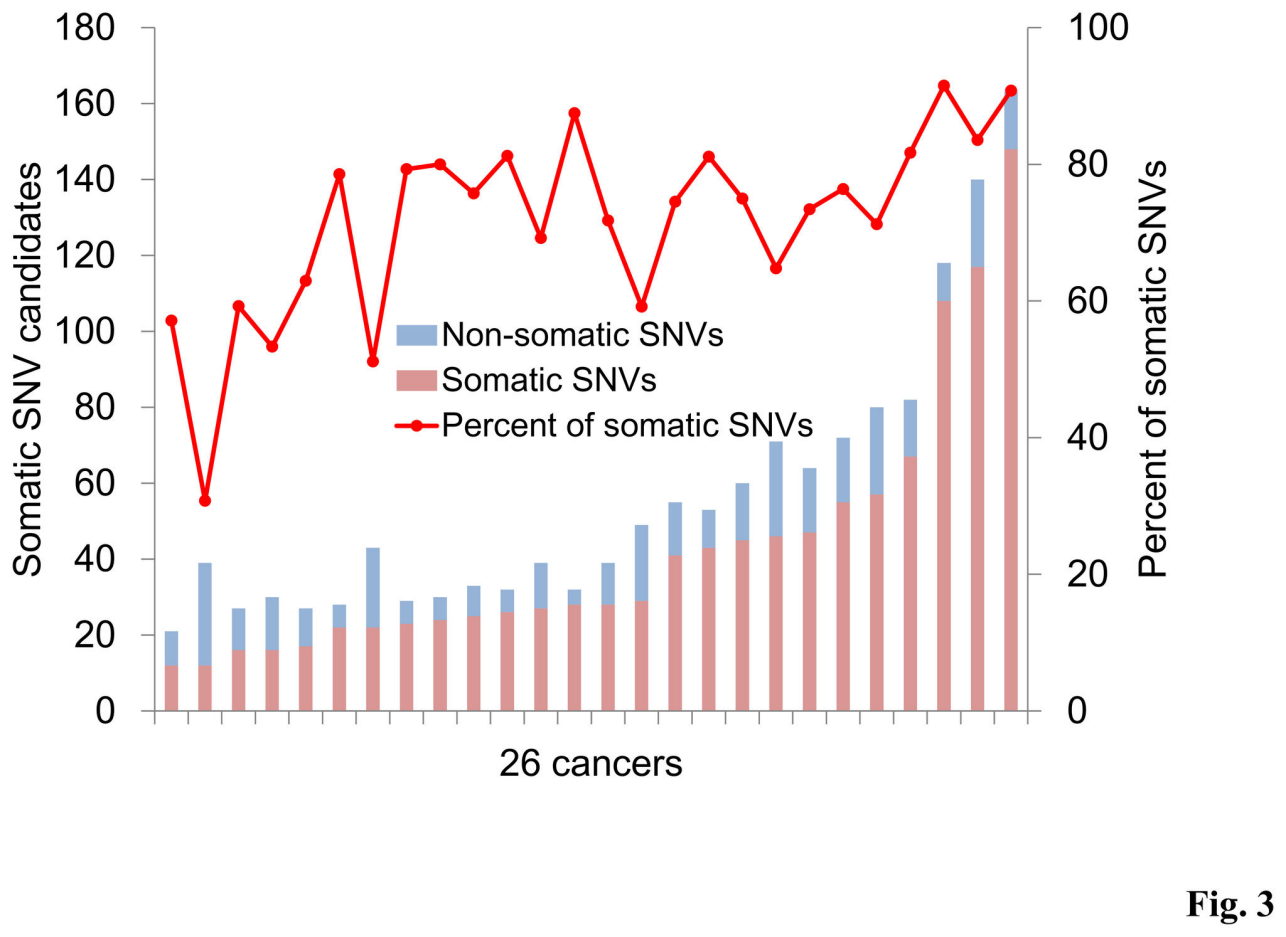
Fidelity of the germline SNV detection in cancer exome analysis. Somatic SNV candidates were identified by using 26 cancer exomes and each normal counterpart. Correct somatic SNVs and false positives were shown in pink and blue bars, respectively. The 26 cancers used for the analysis were sorted by the increasing total number of SNVs (x-axis).

In the aforementioned analysis, we discriminated germline variants using the normal tissue counterparts. A number of SNVs initially identified as somatic were also found to be present in normal tissues, thus, were false positive calls under the validations by visual inspection of the mapped sequences and Sanger sequencing. To examine the cause of this problem, we inspected the errors in randomly selected 26 cancers and their normal tissues. On average in each cancer, twenty-five percent of somatic SNV candidates were found to be false positive (Figure **3**). In these cases, the sequence coverage and quality of the normal counterpart were not sufficient. Indeed, the sequences supporting each SNV and these qualities were significantly diverged between the cancer and normal tissues. Although we increased the total number of reads in the normal tissues, it was difficult in practice to cover all of the genomic positions (Figure S4 in File **S1**). A summary of the germline SNV validations is shown in Table S5 in File **S1**.

However, we noticed that some were correctly identified as germline SNVs in external reference exomes. Twenty-five exomes allowed us to exclude eight false positive calls in each cancer. This raised the possibility that the SNVs from the other patients may be used as surrogates to increase the depth and quality of the sequencing.

### Excluding germline SNVs by considering mutual overlaps of other persons’ exomes

**Figure 4:**
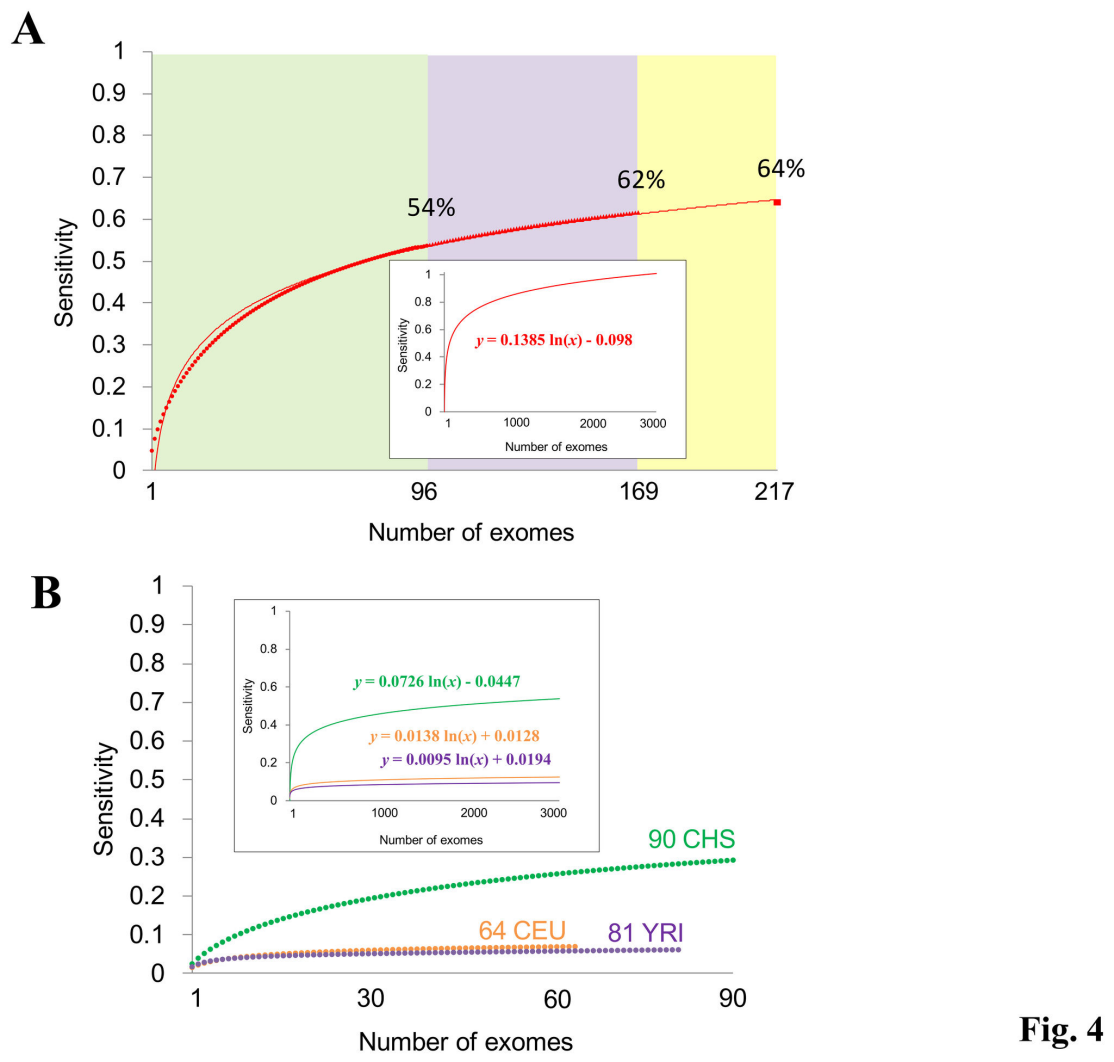
Discriminative powers of detecting germline SNVs using external references. (**A**) The power of detecting germline SNVs considering mutual overlap between other Japanese individuals. Sensitivity represents the proportion of germline SNVs correctly detected. The datasets used to exclude the germline SNVs are shown on the x axis. The inset represents the extrapolation of the graph. Fitting curve of the graph is also shown. (**B**) Discriminative powers of three different ethnic groups for the germline SNVs in 97 Japanese cancers. Sensitivities for detecting germline SNVs are shown by the following colors; green: Chinese; purple: Yoruba; orange: Caucasian.

To further test this possibility, we examined whether cancer exome analyses would be possible without sequencing of the normal tissue counterpart of each cancer. First, we evaluated the extent to which the germline SNVs could be discriminated using external exomes. For this purpose, we used the 97 paired cancer-normal exome datasets for the validation dataset. We found that we could detect 54% of the germline SNVs by using the 96 normal tissue exomes from the external reference (Figure **4A**). We further expanded the filtration dataset using the externally available 73 Japanese exome data and 48 in-house Japanese exome datasets. Altogether, we were able to remove 64% of the germline SNVs, using a total of 217 Japanese exome datasets from other individuals, without sequencing each cancer’s normal counterpart (Figure **4A**). The extrapolation of the graph also indicated that 1,350 and 2,000 samples would be required to remove 90% and 95% of the germline SNVs, respectively. We expect that such a sample size will be available in near future considering current rapid expansion of the exome analysis.

We further evaluated if the same filtration could be done by solely using cancer exomes. We obtained essentially the same results (Figure S5 in File **S1**). Obvious caveat of this approach is that this would disregard about 3% of somatic SNVs recurrently occurring (Figure S5 in File **S1**, blue). However, as aforementioned, we found that those recurrent SNVs were very rare [15,19] and most of them were derived from dubious somatic SNVs, which were overlooked in the normal tissues. We also consider that most of those recurrent SNVs, if any, can be analyzed separately by sequencing a limited number of normal tissues.

### Filtering out germline SNVs by considering mutual overlaps for different ethnic groups and for rare SNPs

We examined whether SNVs in other ethnic backgrounds could be used as external datasets for the filtration. We obtained exome data from individuals of various ethnic backgrounds from the 1000 Genome Project. We used these exome datasets to exclude the germline SNVs that were identified in the Japanese cancers. We found that the discriminative power was significantly lower compared with exomes from Japanese populations. Therefore, these datasets were not suitable for this purpose (Figure **4B**). We also examined and found that the exomes in each ethnic group were useful to discriminate the germline SNVs in the corresponding group (Figure S6, S7 and Table S6 in File **S1**).

We, then, examined to what extent minor germline variants could be covered with this approach in the Japanese population. We evaluated the sensitivity of the filtration process for the SNVs in the 97 cancers (Figure S8 in File **S1**). We found that 88% of the germline SNVs occurring in more than five percent of the 97 exomes could be detected using the 73 external Japanese datasets. For the SNVs occurring in 1% of the 97 cancers, 19% could be excluded.

### Using the crude dataset to characterize cancer related SNVs and pathways

Taken together, with 217 Japanese exomes used for filtration, 36% of the germline SNVs remained unfiltered. Nevertheless, we considered that it may be still possible to use the crude SNV dataset as a first approximation for identifying and analyzing cancer-related genes and pathway candidates. To validate this idea, we compared the results of enrichment analyses between the crude dataset and the refined somatic SNV datasets, which were generated from the paired cancer-normal exomes.

Most of the putative cancer-related genes and pathways that were identified from the refined dataset were also present in the crude dataset (Tables S7 and S8 in File **S1**). The example of the TNN gene, which was reported as a marker of tumor stroma [40-42], is shown in Figure S9 in File **S1**. In this case, even with the germline SNVs, which were unfiltered in the crude dataset (indicated by black in Figure S9 in File **S1**), the enrichment of somatic SNVs in this domain was statistically significant. In total, nine genes which identified as possessing cancer-related SNVs from the refined dataset were also detected in the crude dataset. On the other hand, two genes from the refined dataset were not represented in the crude dataset. In the pathway analysis, we identified 26 cancer-related pathways which were identified from the refined dataset. In addition, 19 pathways were also represented in the crude dataset as well as the refined dataset. The overlap between the datasets is summarized in Table **3**. It should be noted that statistically enrichment analyses were possible even at the current coverage of the filter dataset. With the expanded external dataset, it would be more practical to subject the candidates to the results of Sanger sequencing validations as well as removing remaining germline SNVs.

**Table 3:** Comparison of the results in the enrichment analyses between the crude and refined dataset.

	Number of identified genes/pathways		
	Crude^*^	Refined^†^	Overlap^‡^
Genes	16	11	9
Pathways	23	26	19

^*^ Identified using the crude dataset.

^†^ Identified using the refined dataset.

^‡^ Significant in both crude and refined datasets.

### Identification of prognosis related genes by using the crude dataset

**Figure 5:**
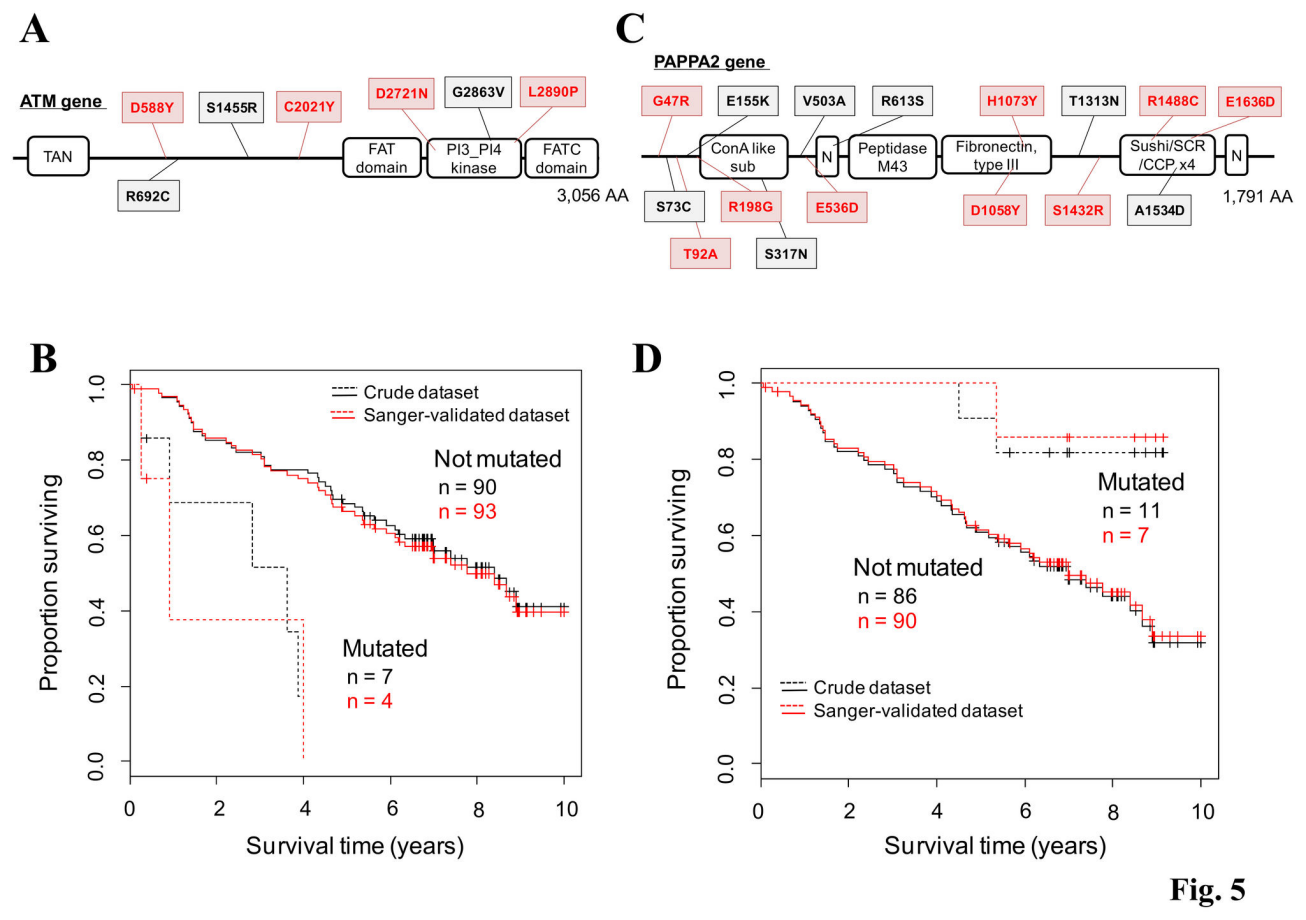
Identification of the putative prognosis-related genes. (**A**) SNVs in the ATM gene. The SNVs that were identified in the initial screening and those remaining after the Sanger sequencing validation of the normal-tissue counterpart were shown in black and red, respectively. TAN: Telomere-length maintenance and DNA damage repair; PI3_PI4 kinase: Phosphatidylinositol 3-/4-kinase, catalytic. (**B**) Survival analysis of patients with and without ATM SNVs. The datasets before and after the Sanger sequencing validation are represented by black and red lines, respectively. Statistical significance was calculated using a log-rank test (*P* < 0.05). Note that the survival differences for individuals with SNVs in the non-Sanger-validated dataset were significant before the Sanger validation. (**C**, **D**) Results of a similar analysis as that described in A and B for the PAPPA2 gene. In this case, the patients with the SNVs showed better prognoses. ConA like sub: Concanavalin A-like lectin/glucanase, subgroup; N: Notch dimain; Peptidase M43: Peptidase M43, pregnancy-associated plasma-A.

As one of the most important objectives of the cancer exome studies, we investigated whether mutations affecting cancer prognoses can be identified by using crude dataset (Table S9 and Figure S10 in File **S1**). In the Kaplan-Meier analysis, seven patients who carried SNVs in the ATM gene (Figure **5A**) showed statistically significant poor prognoses (*P* = 9.6e-6, Figure **5B**). Three SNVs in the ATM gene were significantly enriched in the the phosphatidylinositol 3-/4-kinase catalytic domain (*P* = 0.014). ATM senses DNA damage and phosphorylates TP53, which, in turn, invokes various cellular responses, such as DNA repair, growth arrest and apoptosis, and collectively prevents cancer progression (Figure S11 in File **S1**) [43,44].

We also examined whether other frequently mutated genes were associated with better or worse prognoses. We found that patients with PAPPA2 mutations showed prolonged survival times (*P* = 0.026, Figure 5C and D). PAPPA2 proteolyzes IGFBP5 [45,46], which is an inhibitory factor for IGFs [47]. Mutations in the PAPPA2 gene may result in the accumulation of IGFBP5, and the resulting decrease in IGF signaling may impair the proliferation of cancer cells [48]. Again, it should be noted that for both the ATM and PAPPA2 genes, the statistical significance of the prognostic difference persisted both before (black line) and after (red line) the remaining germline mutations were removed, which was validated by Sanger sequencing (Figure 5B, D and Table S10 in File **S1**). 

## Conclusions

We have identified and characterized the SNVs in lung adenocarcinoma in a Japanese population. Further biological evaluations of the discovered SNVs will be described elsewhere. In particular, information of transcriptome and epigenome should be important for further analyses of cancer genomes, as they would shed new lights on the cancer biology (Table **S1**) [49]. In this study, we also presented a useful approach for the analysis of cancer exomes, without the need to sequence the normal tissue counterpart. We believe that the approach not only lowers the barriers in cost, time and data fidelity in the exome analysis, but also enables exome analysis of archive samples, for which normal tissue counterparts are not always available. 

## Materials and Methods

### Ethics statement

All of the samples were collected by following the protocol (and written informed consent) which were approved by Ethical Committee in National Cancer Center, Japan (Correspondence to: Katsuya Tsuchihara; ktsuchih@east.ncc.go.jp).

### Case selection and DNA preparation

All of the tissue materials were obtained from Japanese lung adenocarcinoma patients with the appropriate informed consent. Surgically resected primary lung adenocarcinoma samples with lengthwise dimensions in excess of 3 cm were selected. Data on the 52 patients who had relapses and other clinical information about the 97 cases are shown in Table S11 in File **S1**. All 97 cancer and normal tissues were extracted from methanol-fixed samples by laser capture microdissection. DNA purification was performed using an EZ1 Advanced XL Robotic workstation with EZ1 DNA Tissue Kits (Qiagen).

### Whole-exome sequencing

Using 1 µg of isolated DNA, we prepared exome-sequencing libraries using the SureSelect Target Enrichment System (Agilent Technologies) according to the manufacturer’s protocol. The captured DNA was sequenced by the illumina Genome Analyzer IIx platform (Illumina), yielding 76-base paired-end reads.

### Somatic SNV detection

The methods that were used to detect the SNVs, including BWA, SAMtools [50] and GATK, are shown in Figure S2 in File S1. Using data from NCBI dbSNP build 132 and one Japanese genome [51], major germline SNVs were excluded. In addition, rare germline SNVs were discarded using 97 exomes from normal tissue counterparts, 73 Japanese exomes provided from the 1000 Genomes Project (the phase1 exome data, 20110521) and 48 in-house Japanese exomes. We also validated a portion of the SNV datasets by the Sanger sequencing of cancer tissues and their normal tissue counterparts (Figure S12 in File **S1**).

### Identification of highly mutated genes

We detected genes which were significantly enriched with SNVs by calculating the expected number of cancers with SNVs in the gene. The length of total CDS regions was represented in *N* (approximately 30.8 M bases). When one patient harbored total of *m* SNVs, the probability that the patient harbors SNVs in the gene *t* (length: *n*) was calculated as *P*:

$$P_{m,t,n}=1-{\left(1-\frac{m}{N}\right)}^{n}$$

The sum of *P* in 97 cancers was represented in the expected number of cancers with SNVs in the gene *t*. The p-values of the observed number were calculated by the Poisson probability function using R ppois.

### Statistical approach to enrichment analyses

To examine the enrichment of mutations in functional protein domains, we mapped the SNVs to domains using InterProScan [52] and assigned them to the Catalogue of Somatic Mutations in Cancer (COSMIC). We analyzed the enrichment of the SNVs in the same domains as the mutations that were provided by the COSMIC. The p-values for the observed mutations in these domains were calculated using their hypergeometric distributions (R phyper). Briefly, the domains in which the SNVs were enriched statistically significantly than the expected number of SNVs in the given length of the domain were selected. For estimating the expected number, the total number of the SNVs belonging to the gene was divided by the gene length. For this analysis, we used genes harboring five or more SNVs in the coding region and three or more SNVs in the domain.

We assigned SNVs to pathways as described by the Kyoto Encyclopedia of Genes and Genomes (KEGG) and calculated the enrichments of the SNVs in the pathways. The mutation rate *M* represented the ratio of the average number of mutated genes to the total number of genes (17,175) that were used in our study. The expected value for the number of cancers with SNVs in pathway *t* was designated *λ* and calculated from the mutation rate *M* and the number of genes in the pathway *n* as follows:

$$\lambda_{t,n}=\left\{1-{\left(1-M\right)}^{n}\right\}\times 97$$

The p-value for the observed number of cancers with SNVs in pathway *t* was calculated by the Poisson probability function using R ppois.

### Estimate of discriminative power for exclusion of germline SNVs by considering mutual overlaps

We estimated the discriminative power for the exclusion of germline SNVs by considering those from other non-cancerous exomes. Germline SNVs from 97 paired tumor-normal exomes were used as reference datasets. Up to 217 samples (96 normal tissue exomes from others and 121 additional Japanese exomes) were randomly selected, and their sensitivities and specificities for detecting the germline SNVs were detected by taking the averages of either all of the combinations or a subset of approximately 10,000 combinations. We also estimated the discriminative power with data from the 1000 Genomes Project for four ethnic groups (73 JPT, 90 CHS, 81 YRI and 64 CEU) using similar trials. Whole-exome sequences (the phase1 exome data, 20110521) were obtained from the ftp site in the 1000 Genomes Project.

### Kaplan-Meier curves

The Kaplan-Meier method was used to test the relations of the observed mutations to survival time, and calculations were performed using the R software package. Changes in survival rates that were correlated with SNVs were examined using the log-rank test (R survdiff).

### Data access

Full raw datasets will be shared with researchers upon request. The information of somatic mutations at the respective genomic coordinates has been provided in Table **S2**.

## Supporting Information

File S1 **Figures S1 to S12 and Tables S3 to S11 are included.**
(PDF)

Table S1 **The comparison of our dataset with the other different study.** We provided the comparison of our dataset with the genes identified in the other different study with transcriptome and epigenome data in lung cancers.
(XLSX)

Table S2 **The list of somatic mutations identified from the refined dataset.** All mutations described in this table are somatic and non-synonymous mutations.
(XLSX)
