# Supplementary material for: Identification and Characterization of Cancer Mutations in Japanese Lung Adenocarcinoma without Sequencing of Normal Tissue Counterparts
Source: PLoS One. 2013 Sep 12;8(9):e73484. doi: 10.1371/journal.pone.0073484 (PMC3772023; doi:10.1371/journal.pone.0073484)
Supplement: File S1 — Figures S1 to S12 and Tables S3 to S11 are included. (PDF) [file pone.0073484.s001.pdf]

SUPPORTING FIGURES

	AD001 (cancer)	Avg. per lane	
		97 cancers	97 normal counterparts
Mapped tags (pair)	34,059,742	32,997,883	33,316,775
Depth (avg.)	76.1	74.1	63.7
5× coverage	0.92	0.93	0.94

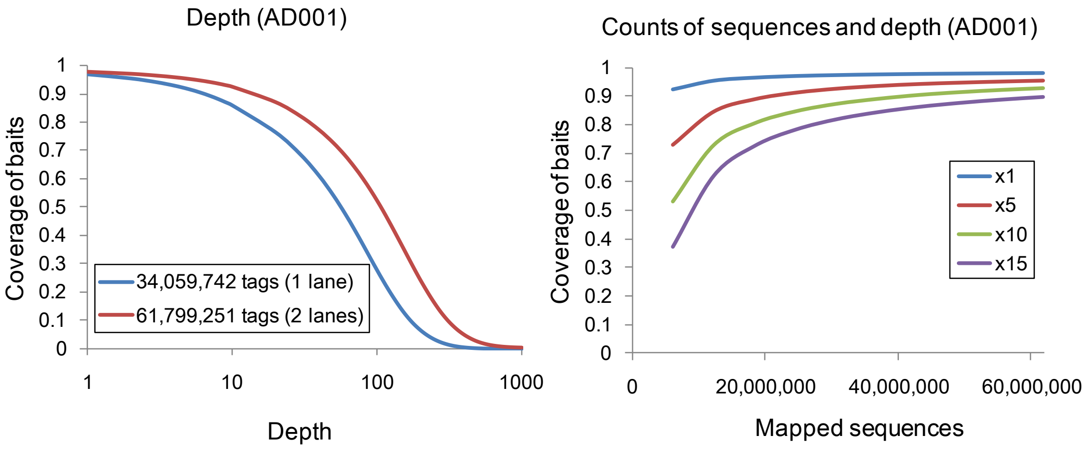

Figure S1. Depth and coverage of whole-exome sequencing. In the upper panel, sequence tags that were mapped to the human genome (UCSC hg19) with sufficient quality (mapping quality = 60) were counted. The average depth and target coverage were calculated based on the exon capture baits. In the lower panel, the depth (left) and sequence counts (right) are shown with the bait coverage for the case AD001.

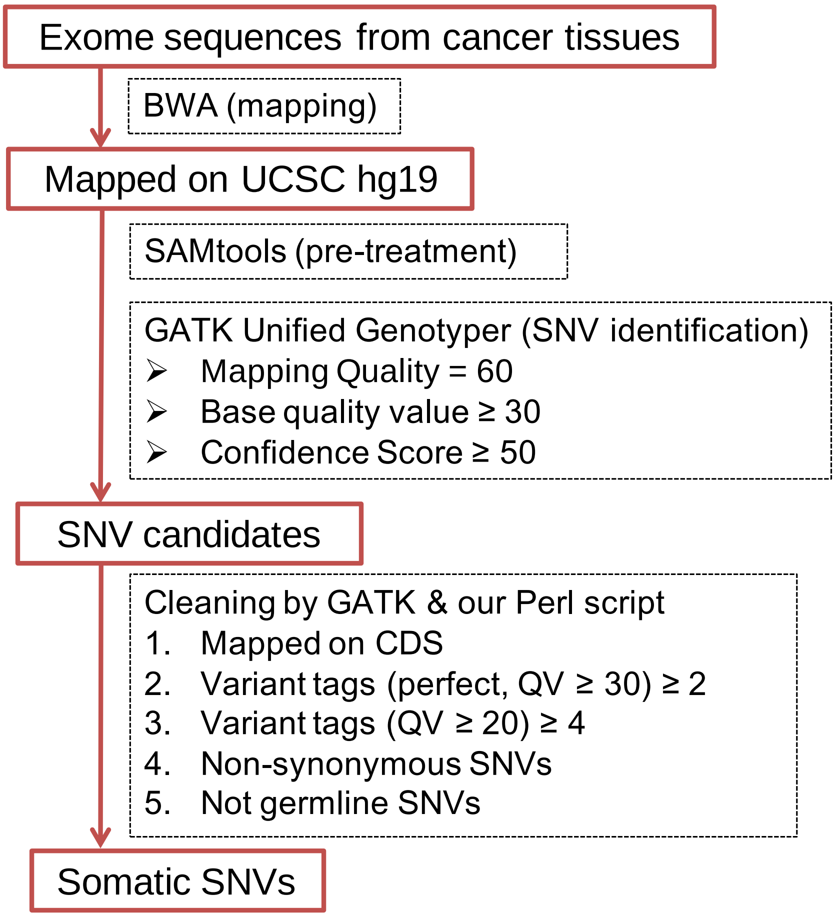

Figure S2. A work flow used to detect SNVs. The paired-end sequences were mapped to the human reference genome (UCSC hg19) using the Burrows-Wheeler Aligner (BWA) [1] in the SAM format. The SNVs were identified by the Genome Analysis Toolkit (GATK) Unified Genotyper [2,3] after the sequences were sorted, PCR duplicates were removed and indexing was performed using SAMtools [4]. Using our Perl scripts, the SNVs were mapped in the CDS regions of 21,730 genes and screened under the following conditions: 1) ≥ 2 tags with a base quality value ≥ 30 mapped perfectly with the exception of each variant and 2) 4× or more with a base quality value ≥ 20 at the position of the SNVs. The NCBI dbSNP [5] build 132 (with the exception of OMIM-CURATED-RECORDS with creating build 132) and one Japanese genome [6] were used to exclude the known SNPs. The non-synonymous SNVs were classified as novel SNVs with changes in the protein sequences. Finally, somatic SNVs were detected only in cancer tissues and showed no evidence of variation in normal tissues.

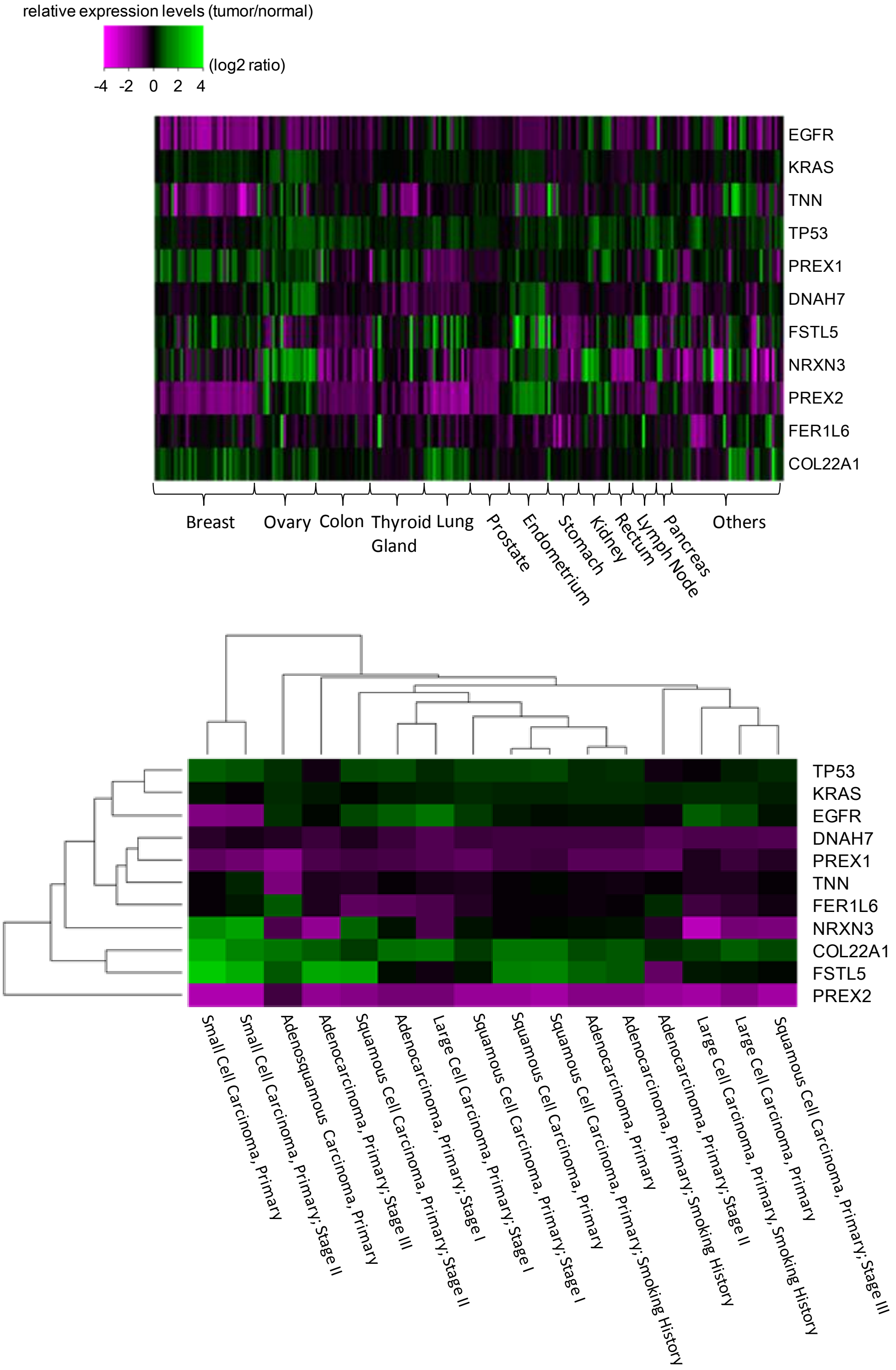

Figure S3. Expression of cancer-related gene candidates. Gene expression profiles of genes detected by statistical enrichment analysis (see **Table 1**) using microarray data provided by GeneLogic. The heatmaps show fold changes in the expression levels between the cancer and normal tissues in 219 cancer types (upper) and lung cancers (lower), which were categorized as indicated in the bottom margin.

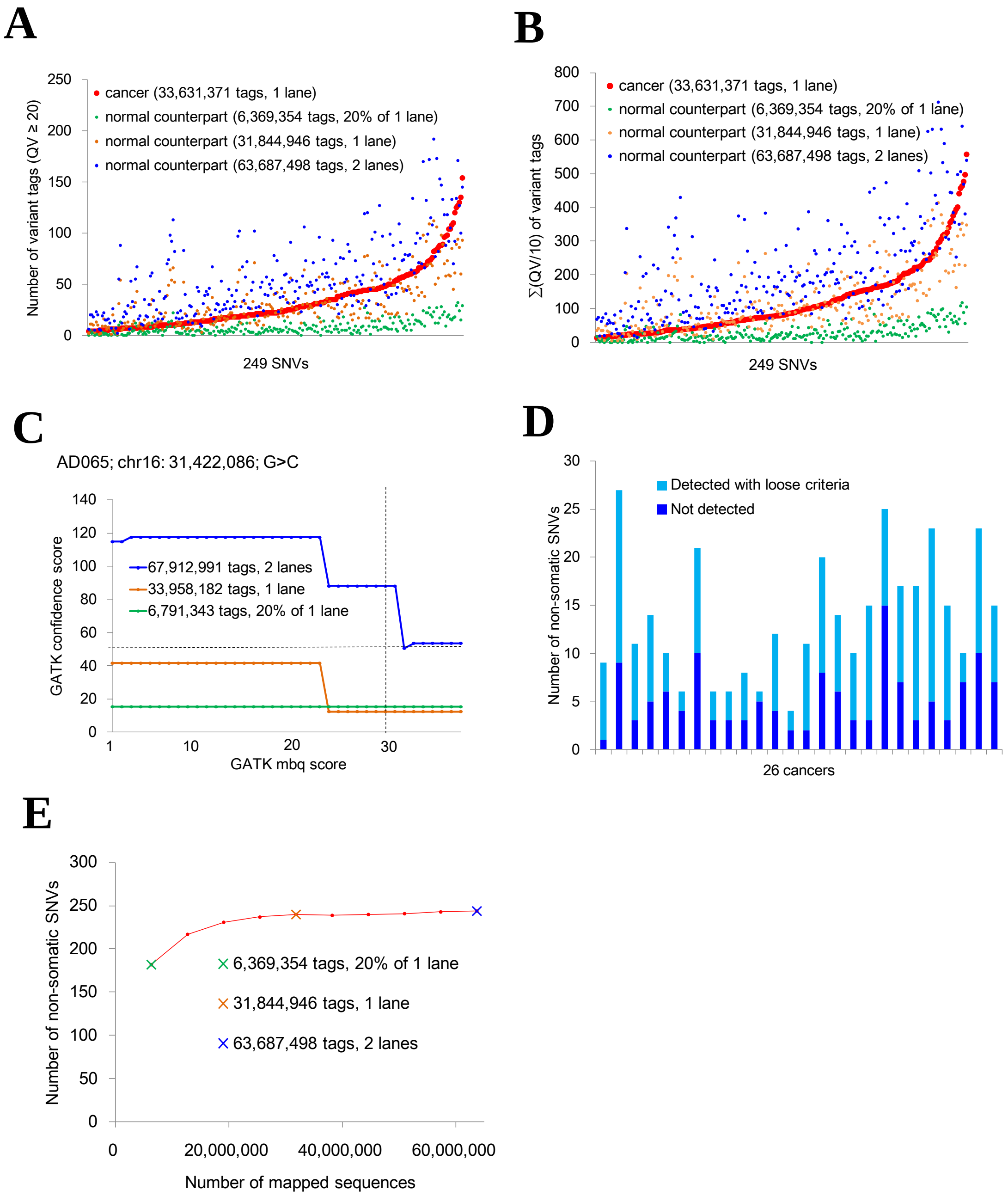

Figure S4. Fidelity of the germline SNV detection in cancer exome analysis. (**A**, **B**) Diversities of sequence depth and base quality value in cancer samples and normal counterparts. Number of sequences (**A**) and cumulated quality value (QV; **B**) supporting the variants in the respective datasets are plotted for the 249 SNVs identified in the case AD021. The SNVs were sorted by the sequence depths of the cancer sequences. (**C**) Sequence depths and GATK scores of an example of germline SNVs by the indicated dataset. A single germline mutation on chromosome 16 in the case AD065 is shown. mbq score of 30 (x-axis) and confidence score of 50 (y-axis) in GATK, represented by broken

lines, are frequently used cut-offs. (**D**) Germline SNVs and errors remaining in the datasets of somatic SNV candidates that were identified in 26 cancers. When loose criteria were used to identify SNVs in the normal counterparts, the germline SNVs were either not detected or only partially detected (shown in blue and light blue, respectively). Order of the 26 cancers in x-axis is as in **Fig. 3**. (**E**) The number of SNVs that were detected in normal counterparts at their respective sequence depths. The graph shows the number of germline SNVs in normal counterparts at the indicated sequence depths (green cross; randomly selected 20% of one lane; orange and blue crosses; one and two lanes, respectively) for the case AD021.

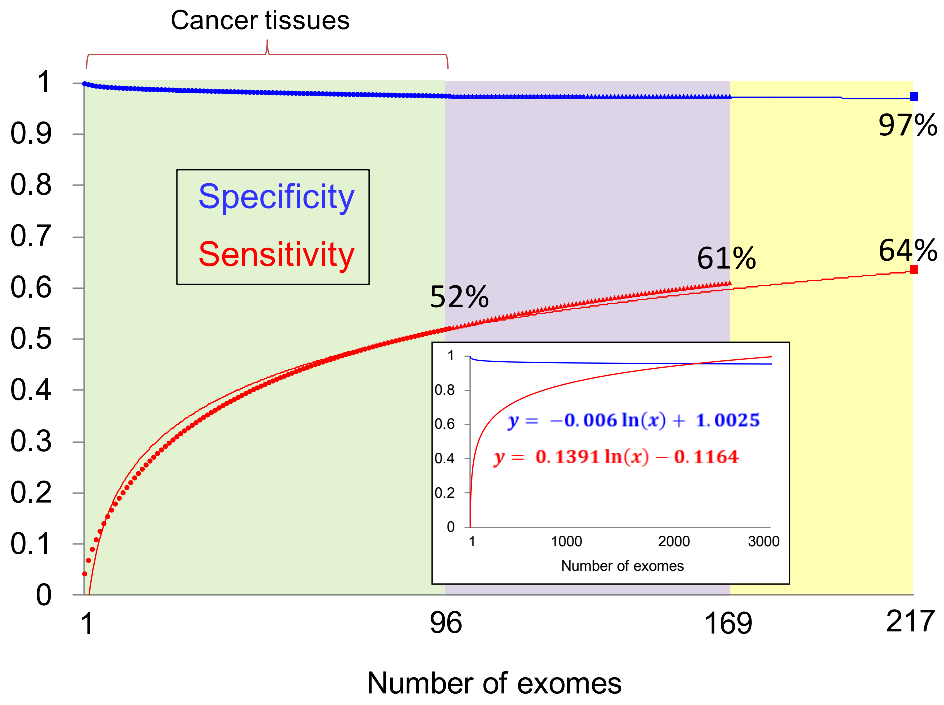

Figure S5. Sensitivity and specificity of detecting germline SNVs considering overlap with other individuals. The SNV datasets of 96 cancers collected in this study and 121 unrelated Japanese donors were used to exclude the germline SNVs. Similarly to **Fig. 4A**, extrapolation of the graph and the fitting curves are shown in the inset.

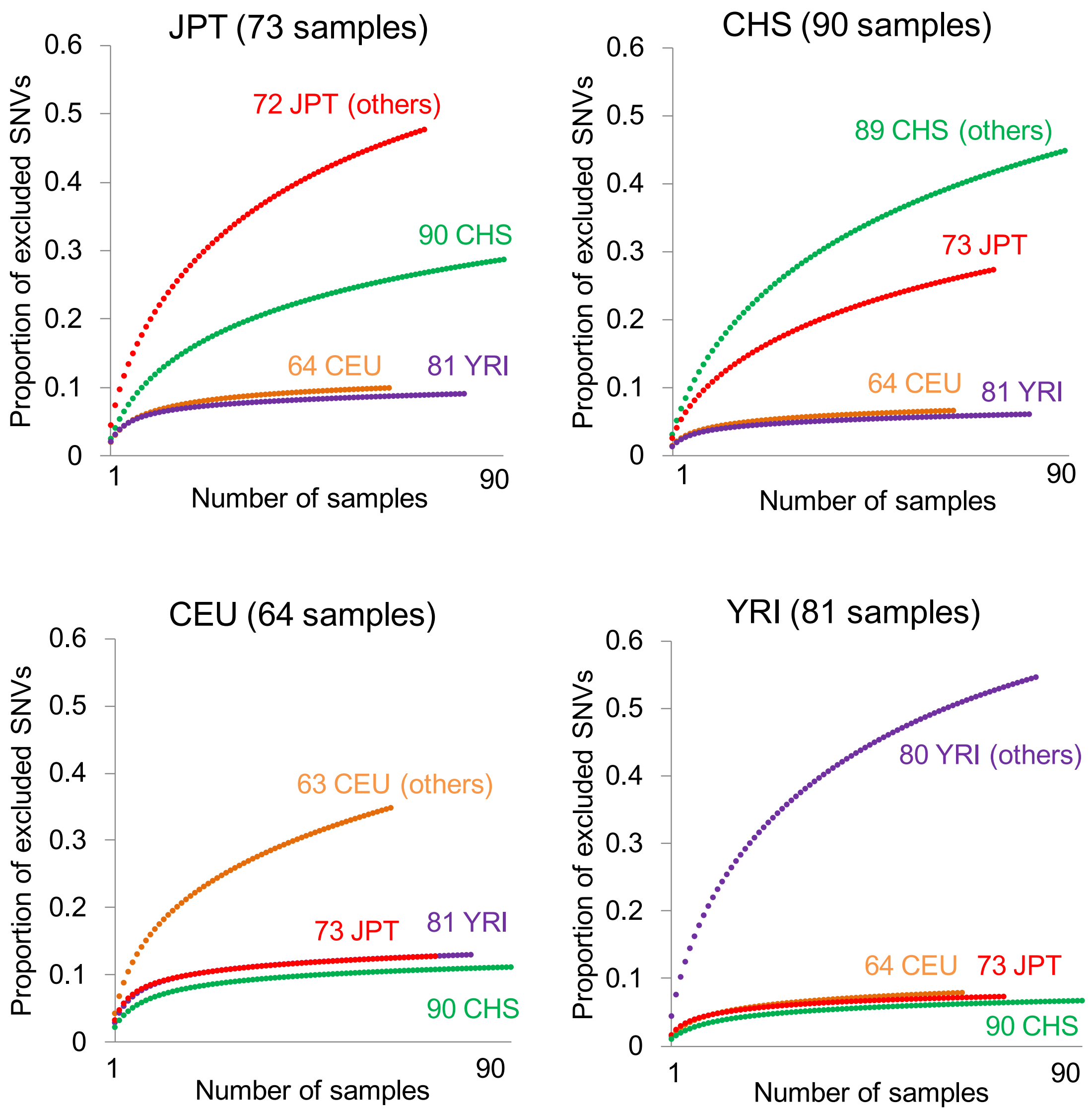

Figure S6. Frequency of germline SNVs overlapping between ethnic groups. The following four ethnic groups are shown in the colors listed: JPT (red): Japanese; CHS (green): Chinese; YRI (purple): Yoruba; CEU (orange): Caucasian. For each ethnic group, the proportions of SNVs that mutually overlap with the other groups are shown.

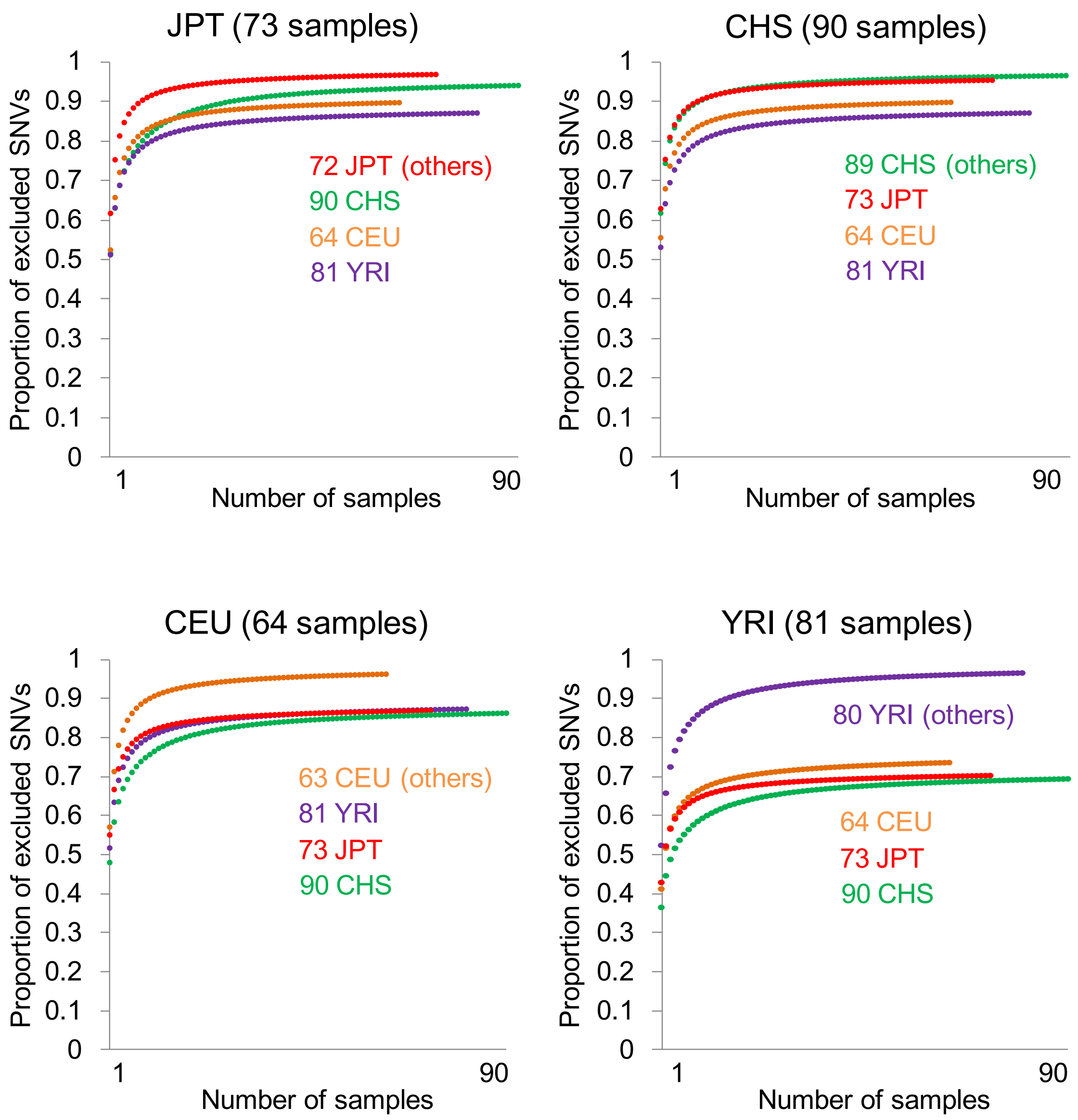

Figure S7. The results of the similar analysis shown in **Fig. S6**. The datasets were used without removing publicly available SNPs (dbSNP build 132 and one Japanese genome).

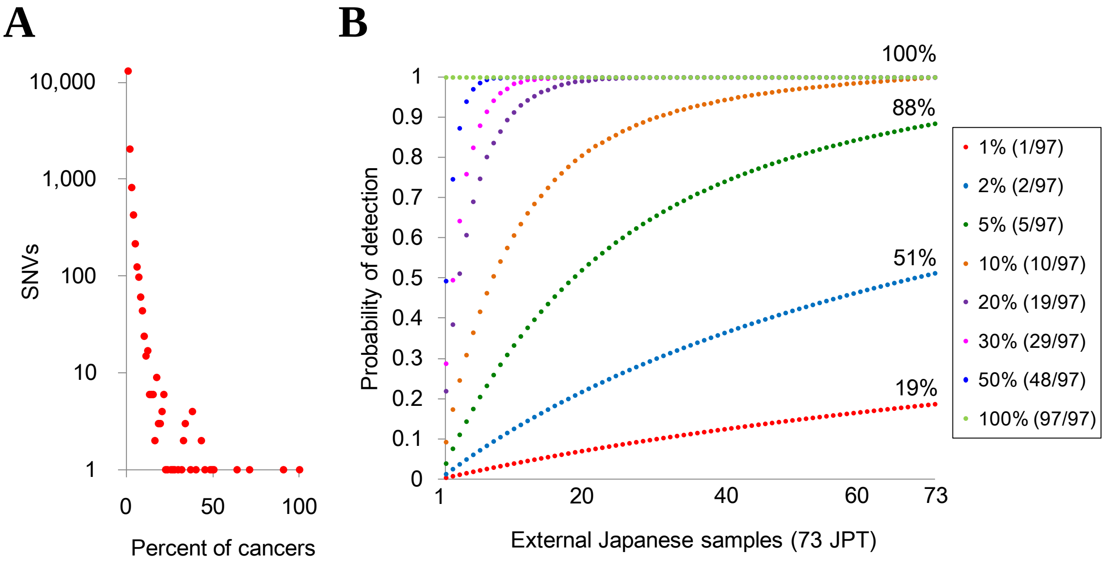

Figure S8. Germline SNVs in the Japanese population. **(A)** Frequencies of rare germline SNVs in 97 cancers. Rare germline SNVs were detected in the 97 paired tumor-normal sequences after excluding public SNPs. **(B)** Probabilities of the detecting rare germline SNVs at each frequency. The numbers in the margin indicate the probabilities of detecting rare germline SNVs in the 97 cancers, using the 73 external Japanese exomes.

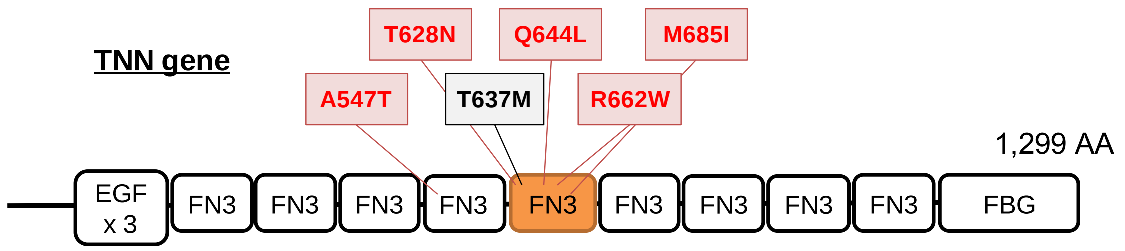

Figure S9. SNVs in the TNN genes. The SNVs from the crude dataset and those remaining after the validations (the refined dataset) were shown in the black and red letter, respectively (also see **Table S7**). EGF: EGF-like domain; FN3: Fibronectin, type III domain; FBG: Fibrinogen, alpha/beta/gamma chain, C-terminal globular domain.

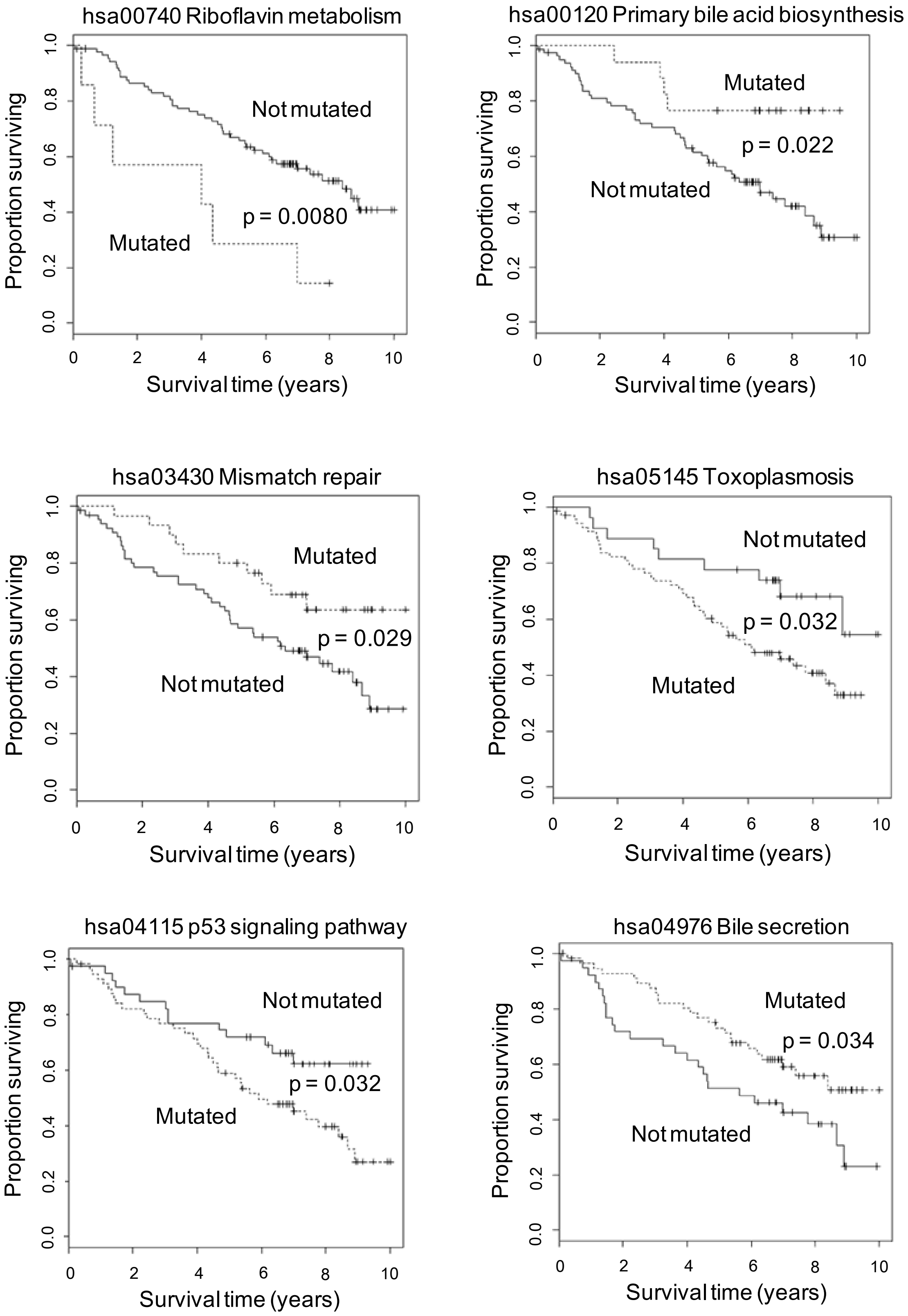
hsa00740 Riboflavin metabolism
Not mutated
p = 0.0080
Mutated
Proportion surviving
Survival time (years)
hsa00120 Primary bile acid biosynthesis
Mutated
p = 0.022
Not mutated
Proportion surviving
Survival time (years)
hsa03430 Mismatch repair
Mutated
p = 0.029
Not mutated
Proportion surviving
Survival time (years)
hsa05145 Toxoplasmosis
Not mutated
p = 0.032
Mutated
Proportion surviving
Survival time (years)
hsa04115 p53 signaling pathway
Not mutated
p = 0.032
Mutated
Proportion surviving
Survival time (years)
hsa04976 Bile secretion
Mutated
p = 0.034
Not mutated
Proportion surviving
Survival time (years)

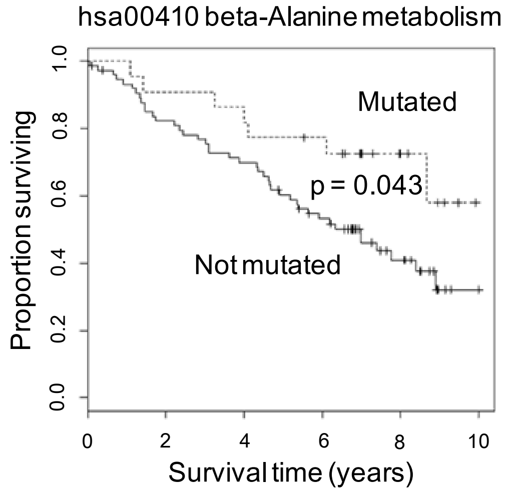

Figure S10. Identified putative prognosis-related pathways using the crude dataset of somatic SNVs. The overall survival times were compared between cases with and without SNVs in each pathway. Seven pathways that were described in the KEGG database[7] were significantly related to prognosis ($P < 0.05$).

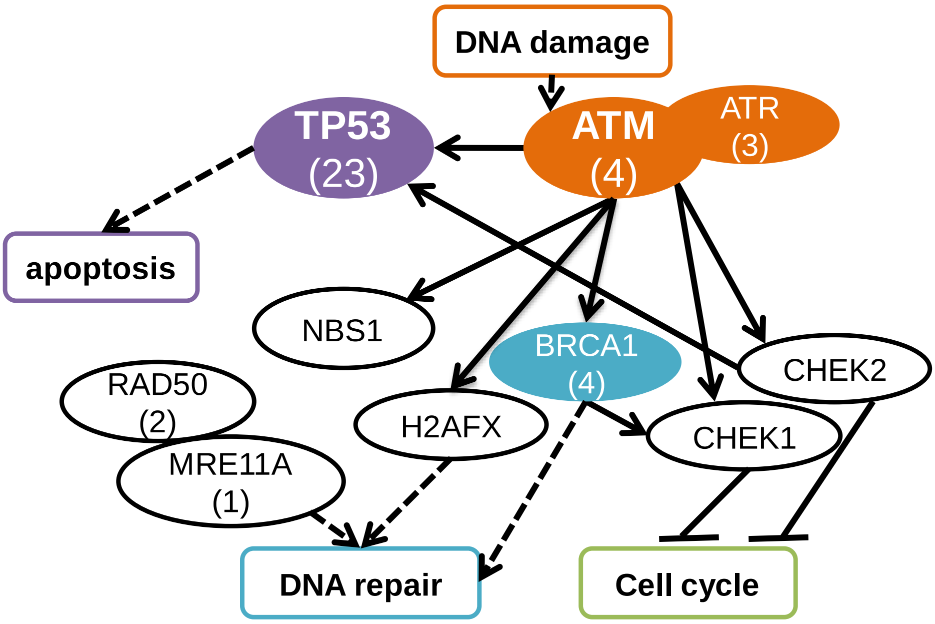

Figure S11. The DNA damage-response pathway containing the ATM, TP53 and other cancer-related genes. The numbers of cancers with SNVs in each gene are provided in parentheses. These results were from the Sanger-validated dataset.

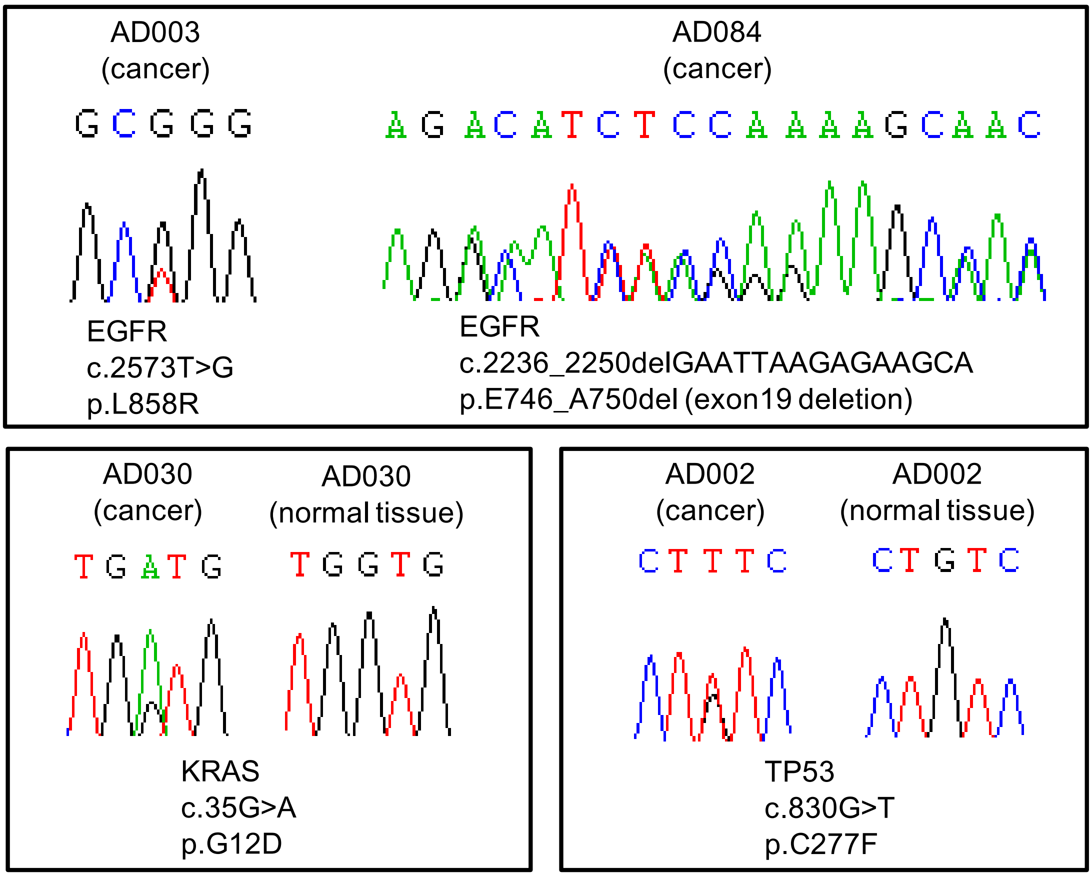

Figure S12. Validation by Sanger sequencing. Using Sanger sequencing, SNVs in some of the genes were validated as either somatic or cancer-specific. A few example chromatograms are shown.

SUPPORTING TABLES

Table S3. Somatic SNVs in 97 cancer exomes

Positions of SNVs	Total	9,604
	Average	99.8
	Range	13 – 1,331
Genes with SNVs	Total	5,908
	Average	96.1
	Range	13 – 1,202

Table S4. List of the highly mutated genes

Gene	Number of cancers with SNVs	P-value*
EGFR	37	3.0e-39
TP53	23	5.9e-30
CSMD3	14	9.1e-6
SPTA1	12	2.8e-6
HYDIN	12	0.0027
MUC17	11	0.0021
XIRP2	10	0.0040
USH2A	10	0.023

*≥10 cancers and $P < 0.05$ (also see Materials and Methods).

Table S5. Germline SNV detection statistics on 26 cancer-normal paired exomes

Average number of SNVs on 26cancers[*]		312.5
Somatic SNVs[†]		42.3
Detected in the normal counterpart		256.5
Detected in the normal counterpart with loose criteria[‡,§]		264.9
Detected using the normal counterpart and 25 other normal tissues[§]		264.2
Average number of germline SNVs not detected even with loose criteria[‡,§] (range)		5.3 (1 - 15)
Average depth (QV ≥ 20) of 249 germline SNVs in the case AD021 (range)	Cancer (1 lane)	32.5 (4 - 154)
	Normal (20% of 1 lane)	7.7 (0 - 32)
	Normal (1 lane)	32.2 (1 - 112)
	Normal (2 lanes)	55.1 (2 - 192)

[*]After removing dbSNP build 132 and one Japanese genome.

[†] Manually checked on Integrated Genome Viewer (IGV) [8,9].

[‡] The confidence score ≥ 10 and the mbq score of 10 at GATK.

[§]Not including two severe conditions in the analytic pipeline shown in **Fig. S2**; variant tags (QV ≥ 20) ≥ 4, variant tags (perfect) ≥ 2.

Table S6. dbSNP coverage in each population

		Number of SNPs (dbSNP)*			
		JPT	CHS	CEU	YRI
at least 1 person		3,270	2,718	6,251	6,763
Percent of population	5%	712	704	1,762	2,394
	10%	507	443	799	1163
	20%	277	162	376	439
	30%	208	134	229	278
	100%	887	795	709	738

*In total, dbSNP build 132 contains 232,861 non-synonymous SNPs within CDS regions.

Table S7. List of the identified possible cancer-related genes using the crude dataset

Gene	Domain	Number of SNVs		P-value*
		Domain	Gene	
EGFR	IPR001245:Serine-threonine/tyrosine-protein kinase	35	39	7.1e-21†
TNN	IPR003961:Fibronectin, type III	5	6	3.5e-6†
KRAS	IPR001806:Ras GTPase	6	7	8.0e-6†
COL4A6	IPR008160:Collagen triple helix repeat	3	5	0.00031
TP53	IPR008967:p53-like transcription factor, DNA-binding	20	24	0.00032†
LRRK2	IPR011046:WD40 repeat-like-containing domain	5	7	0.0017
PREX1	IPR000219:Dbl homology (DH) domain	4	6	0.0020†
ANK3	IPR020683:Ankyrin repeat-containing domain	4	11	0.0079
UNC5B	IPR003599:Immunoglobulin subtype	3	5	0.0087
PREX2	IPR001849:Pleckstrin homology	3	7	0.011†
DNAH7	IPR004273:Dynein heavy chain	5	9	0.011†
SLC26A4	IPR002645:Sulphate transporter/antisigma-factor antagonist STAS	5	7	0.011
NRXN3	IPR008985:Concanavalin A-like lectin/glucanase	5	8	0.014†
ATM	IPR000403:Phosphatidylinositol 3-/4-kinase, catalytic	3	7	0.014
COL22A1	IPR008985:Concanavalin A-like lectin/glucanase	3	6	0.015†
ABCC12	IPR003439:ABC transporter-like	4	7	0.020

*$P < 0.02$.

†Significant in the refined dataset as well ($P < 0.02$).

Table S8. List of the identified possible cancer-related pathways using the crude dataset

KEGG ID	Pathway definition	Number of cancers with SNVs	P-value*
hsa04320	Dorso-ventral axis formation	54	2.2e-8†
hsa05213	Endometrial cancer	76	3.1e-6†
hsa02010	ABC transporters	65	1.3e-5†
hsa05219	Bladder cancer	64	2.3e-5†
hsa05223	Non-small cell lung cancer	74	2.4e-5†
hsa04977	Vitamin digestion and absorption	42	0.00010†
hsa05214	Glioma	75	0.00049†
hsa04512	ECM-receptor interaction	82	0.0020†
hsa05218	Melanoma	75	0.0023†
hsa05212	Pancreatic cancer	74	0.0033†
hsa04520	Adherens junction	73	0.0039†
hsa05216	Thyroid cancer	41	0.0069†
hsa00061	Fatty acid biosynthesis	13	0.013
hsa05222	Small cell lung cancer	77	0.015†
hsa05215	Prostate cancer	78	0.017†
hsa00524	Butirosin and neomycin biosynthesis	11	0.020
hsa05412	Arrhythmogenic right ventricular cardiomyopathy (ARVC)	66	0.022†
hsa05217	Basal cell carcinoma	58	0.025†
hsa05210	Colorectal cancer	63	0.027†
hsa05146	Amoebiasis	82	0.029
Hsa04012	ErbB signaling pathway	75	0.034†
hsa00300	Lysine biosynthesis	7	0.045
hsa05120	Epithelial cell signaling in Helicobacter pylori infection	63	0.045†

*$P < 0.05$.

†Significant in the refined dataset as well ($P < 0.05$).

Table S9. Lists of genes related to prognosis detected from the crude dataset

Genes with SNVs related to poor prognosis

Gene	Number of cancers		P-value*
	SNVs (+)	SNVs (-)	
ATM	7	90	9.6e-6
LAMB1	5	92	0.00021
ANKRD50	5	92	0.00034
BAZ2B	5	92	0.00040
ZNF91	5	92	0.00079
DAB2	5	92	0.0030
SLC44A3	6	91	0.0069
LAMA3	6	91	0.0071
ACADSB	5	92	0.0087
STAB2	7	90	0.014
CCDC108	6	91	0.016
LMTK2	5	92	0.016
STIL	5	92	0.016
VCAN	7	90	0.017
ANKRD30A	8	89	0.024
FN1	14	83	0.025
FAM83H	6	91	0.031
ANKRD55	5	92	0.032
PYGM	5	92	0.037
XPO6	5	92	0.037
KCNA5	5	92	0.039
PRDM2	5	92	0.044
RGS22	5	92	0.046
CDH8	5	92	0.050

Genes with SNVs related to good prognosis

Gene	Number of cancers		P-value*
	SNVs (+)	SNVs (-)	
PAPPA2	11	86	0.026
BMS1	5	92	0.038
TET1	5	92	0.042
FER1L6	5	92	0.046
ELAVL2	5	92	0.049
TMEM2	5	92	0.049

*$P < 0.05$ (log-rank test).

Table S10. Clinical and background information for patients with ATM or PAPPA2 mutations

Gender	Age at the operation	Smoking history	ATM	PAPPA2	Known driver mutation	Overall survival (days)	Relapse free survival (days)	Chemo-thrapy	Lymph node metastasis	Differentiation	Histological subtype
M	65	+	D588Y	None	KRAS G12V	92	92	-	+	low	solid
M	78	+	C2021Y	None	PIK3CA E545K	146	146	-	-	modelately	acinar
M	74	+	D2721N	None	KRAS Q61H	337	25	-	+	modelately	BAC
M	78	+	L2890P	None	KRAS G13D	1,464	614	-	-	modelately	acinar
M	56	+	None	R198G		1,954	777	+	-	modelately	papillary
M	43	+	None	E536D		2,539	2,539	-	+	low	solid
M	64	+	None	G47R, T92A, H1073Y		3,103	3,103	-	-	well	acinar
M	66	+	None	D1058Y		2,563	2,563	-	-	low	solid
M	66	+	None	S1432R		3,272	3,272	-	-	modelately	papillary
F	65	-	None	R1488C	EGFR L858R	3,345	3,345	-	-	well	BAC
M	75	+	None	E1636D	KRAS G12C	3,103	3,103	-	-	low	acinar

Table S11. Background and clinical information

Number of patients	97
Patient age	
Average (years)	66.6
Median (years)	67
Gender	
Male	52
Female	45
Body Mass Index (BMI)	
Average	22.5
Median	22.6
Smoking history	
Smoker	54
Non-smoker	43
Clinical information	
Lymph node metastasis	38
Vascular invasion	58
Lymphatic invasion	55
Pleural invasion	49
Recurrence	52
Carcinoembryonic antigen (CEA)	
Average	18.1
Median	5.3
Histological subtype	
Papillary	39
Bronchoalveolar carcinoma	28
Solid adenocarcinoma (with mucin)	17
Acinar	13

REFERENCES

1. Li H, Durbin R (2009) Fast and accurate short read alignment with Burrows-Wheeler transform. Bioinformatics 25: 1754-1760.
2. McKenna A, Hanna M, Banks E, Sivachenko A, Cibulskis K, et al. (2010) The Genome Analysis Toolkit: a MapReduce framework for analyzing next-generation DNA sequencing data. Genome Res 20: 1297-1303.
3. DePristo MA, Banks E, Poplin R, Garimella KV, Maguire JR, et al. (2011) A framework for variation discovery and genotyping using next-generation DNA sequencing data. Nat Genet 43: 491-498.
4. Li H, Handsaker B, Wysoker A, Fennell T, Ruan J, et al. (2009) The Sequence Alignment/Map format and SAMtools. Bioinformatics 25: 2078-2079.
5. Sherry ST, Ward MH, Kholodov M, Baker J, Phan L, et al. (2001) dbSNP: the NCBI database of genetic variation. Nucleic Acids Res 29: 308-311.
6. Fujimoto A, Nakagawa H, Hosono N, Nakano K, Abe T, et al. (2010) Whole-genome sequencing and comprehensive variant analysis of a Japanese individual using massively parallel sequencing. Nat Genet 42: 931-936.
7. Kanehisa M, Goto S, Furumichi M, Tanabe M, Hirakawa M (2010) KEGG for representation and analysis of molecular networks involving diseases and drugs. Nucleic Acids Res 38: D355-360.
8. Robinson JT, Thorvaldsdottir H, Winckler W, Guttman M, Lander ES, et al. (2011) Integrative genomics viewer. Nat Biotechnol 29: 24-26.
9. Thorvaldsdottir H, Robinson JT, Mesirov JP (2012) Integrative Genomics Viewer (IGV): high-performance genomics data visualization and exploration. Brief Bioinform.
